# Metformin use and cervical cancer risk in female patients with type 2 diabetes

**DOI:** 10.18632/oncotarget.10934

**Published:** 2016-07-29

**Authors:** Chin-Hsiao Tseng

**Affiliations:** ^1^ Department of Internal Medicine, National Taiwan University College of Medicine, Taipei, Taiwan; ^2^ Division of Endocrinology and Metabolism, Department of Internal Medicine, National Taiwan University Hospital, Taipei, Taiwan; ^3^ Division of Environmental Health and Occupational Medicine of the National Health Research Institutes, Zhunan, Taiwan

**Keywords:** cervical cancer, diabetes mellitus, metformin, Taiwan

## Abstract

This study evaluated whether metformin may affect the risk of cervical cancer. The reimbursement databases of the Taiwan's National Health Insurance were used. Female patients with type 2 diabetes at an onset age of 25-74 years during 1999-2005 and newly treated with metformin (*n*=132971, “ever users of metformin”) or other antidiabetic drugs (*n*=6940, “never users of metformin”) were followed for at least 6 months until December 31, 2011. The treatment effect of metformin (for ever versus never users, and for tertiles of cumulative duration of therapy) was estimated by Cox regression incorporated with the inverse probability of treatment weighting using propensity score. Analyses were also conducted in a 1:1 matched pair cohort based on 8 digits of propensity score. Results showed that the respective numbers of incident cervical cancer in ever users and never users were 438 (0.33%) and 38 (0.55%), with respective incidences of 68.29 and 121.38 per 100,000 person-years. The overall hazard ratio suggested a significantly lower risk in metformin users (0.558, 95% confidence intervals: 0.401-0.778). In tertile analyses, the hazard ratios (95% confidence intervals) for the first (<23.0 months), second (23.0-47.9 months) and third (>47.9 months) tertile of cumulative duration were 1.272 (0.904-1.790), 0.523 (0.366-0.747) and 0.109 (0.070-0.172), respectively. Findings were supported by the analyses in the matched cohort. In conclusion, metformin may significantly reduce the risk of cervical cancer, especially when the cumulative duration is more than 2 years.

## INTRODUCTION

Cervical cancer is the third common cancer and the fourth leading cause of cancer death in women [1]. Most cases (>85%) occur in developing countries and are closely related to the infection of human papillomavirus (HPV) [1]. Vaccines against the most common strains of HPV (types 16 and 18 responsible for 70% of cervical cancer) have been used for its prevention. However, because of the high cost, vaccination programs have not been widely implemented.

Metformin, a cheap and commonly used antidiabetic drug, may inhibit the growth and proliferation of cancer cells including the breast [2], endometrium [3], ovary [4], lung [5], thyroid [6], liver [7], esophagus [8], pancreas [9], stomach [10], colon [8], prostate [11], bladder [12] and leukemic cells [13]. Recent epidemiological studies also support that metformin may reduce the risk of cancers involving the colon [14], bladder [15], breast [16], prostate [17], thyroid [18], endometrium [19], ovary [20], kidney [21] and oral cavity [22].

Whether metformin can reduce the risk of cervical cancer has not been studied. Recent *in vitro* studies provide evidence for a protective role. Activation of the liver kinase B1 (LKB1)-5′ adenosine monophosphate-activated protein kinase (AMPK) pathways by metformin may inhibit the growth of cervical cancer cell lines, through blocking the mammalian target of rapamycin (mTOR) [23], the Wnt/β-catenin [24] and the Forkhead Box M1 (FOXM1) [25] signaling cascades. Metformin may also inhibit the growth of cervical cancer HeLa cells through AMPK-independent pathways by inhibiting the expression of heme oxygenase-1 (a heat shock protein that regulates oxidative stress) via inactivation of Raf-ERK-Nrf2 signaling [26].

This study evaluated whether metformin could reduce cervical cancer risk by using the reimbursement databases of the National Health Insurance (NHI). The dose-response relationship was evaluated by the tertiles of cumulative duration of metformin therapy. To solve the problem of “prevalent user bias” [27], newly diagnosed diabetic patients and incident users of metformin were recruited. To reduce “immortal time bias” (the initial period of follow-up during which the outcome can not occur) [28], patients should have been prescribed antidiabetic drugs for at least two times, and those who were followed up for a short period of time (i.e., <180 days) were excluded. To address the differences in baseline characteristics associated with treatment allocation in non-random observational studies, Cox regression models were created by incorporation with the inverse probability of treatment weighting (IPTW) using propensity score (PS) [29] and analyses were also conducted in a 1:1 matched cohort based on 8 digits of PS [30].

## RESULTS

**Figure 1:**
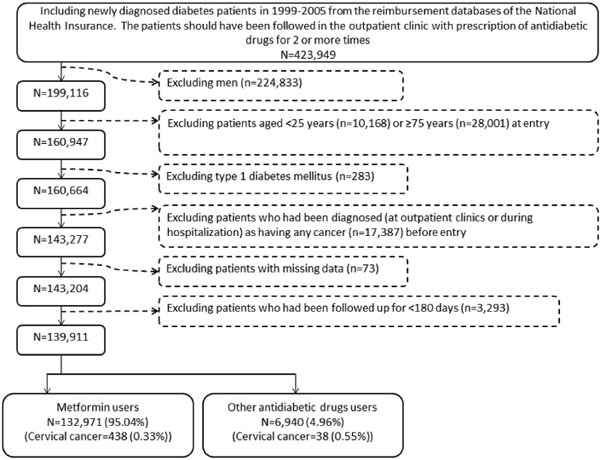
Flowchart showing the procedures in selecting patients into the original cohort

There were 6940 never users and 132971 ever users in the original cohort (Figure 1). All characteristics of the two groups differed significantly in the original cohort, except for peripheral arterial disease and pioglitazone. Ever users were characterized by younger age, higher proportions of obesity, eye disease, dyslipidemia and receiving cervical cancer screening, lower proportions of hypertension, chronic obstructive pulmonary disease, nephropathy, stroke and ischemic heart disease, higher proportion of rosiglitazone use but lower proportions of using other antidiabetic medications (Table 1). However, in the matched cohort, only eye disease and use of sulfonylurea and insulin differed significantly between the two groups (Table 1). While examining the standardized differences, the values for 11 out of the 17 covariates were >10% in the original cohort, but only sulfonylurea and insulin had a value >10% in the matched cohort.

**Table 1:** Baseline characteristics of never users and ever users of metformin in the original cohort and in the propensity score matched cohort

Variables	Original cohort						Matched cohort					
	Never users		Ever users		P*	SD	Never users		Ever users		P*	SD
	*n*	%	*n*	%			*n*	%	*n*	%		
	6940		132971				6940		6940		
Age (years)	60.96±9.95		58.10±10.04		<0.0001	-29.66	60.96±9.95		61.17±9.22		0.0524	4.18
Obesity	222	3.20	7913	5.95	<0.0001	13.23	222	3.20	198	2.85	0.2344	-2.04
Hypertension	5337	76.90	96678	72.71	<0.0001	-10.23	5337	76.91	5377	77.49	0.4183	1.46
Chronic obstructive pulmonary disease	3065	44.16	55444	41.70	<0.0001	-5.53	3065	44.17	3030	43.67	0.5494	-0.90
Nephropathy	1781	25.66	22312	16.78	<0.0001	-23.61	1780	25.65	1769	25.49	0.8305	-1.40
Eye disease	571	8.23	20648	15.53	<0.0001	22.80	571	8.23	489	7.05	0.0088	-4.62
Dyslipidemia	4437	63.93	93249	70.13	<0.0001	13.62	4437	63.94	4364	62.89	0.1983	-1.68
Stroke	1787	25.75	27045	20.34	<0.0001	-13.61	1787	25.75	1777	25.61	0.8459	-0.23
Ischemic heart disease	2886	41.59	49537	37.25	<0.0001	-9.48	2886	41.59	2903	41.84	0.7698	0.41
Peripheral arterial disease	1195	17.22	23160	17.42	0.6710	0.28	1195	17.22	1181	17.02	0.7524	-0.46
Sulfonylurea	5052	72.80	84780	63.76	<0.0001	-16.61	5052	72.81	5316	76.61	<0.0001	11.43
Meglitinide	552	7.95	4627	3.48	<0.0001	-20.47	551	7.94	514	7.41	0.2380	-1.55
Acarbose	828	11.93	6724	5.06	<0.0001	-24.30	827	11.92	768	11.07	0.1163	-3.30
Insulin	481	6.93	2550	1.92	<0.0001	-25.75	480	6.92	318	4.58	<0.0001	-11.80
Pioglitazone	171	2.46	2939	2.21	0.1622	-0.99	171	2.46	146	2.10	0.1555	-2.74
Rosiglitazone	220	3.17	5860	4.41	<0.0001	6.73	220	3.17	201	2.90	0.3470	-1.73
Cervical cancer screening	3263	47.02	67876	51.05	<0.0001	8.29	3263	47.02	3229	46.53	0.5630	-0.78

Age is expressed as mean ± standard deviation; SD: standardized difference; *by Student’s t test for age and by Chi-square test for other variables.

The incidences of cervical cancer by metformin exposure and hazard ratios comparing exposed to unexposed are shown in Table 2. When evaluating the distribution of the incident cases by the tertiles of cumulative duration, there was a trend of decreasing incidence with longer duration of exposure (Table 2). The overall hazard ratio showed a significantly lower risk associated with metformin use. Although the hazard ratio was not significant for the first tertile, those in the second and third tertile suggested a significantly reduced risk in the original cohort (Table 2). The results derived from the matched cohort were very similar to the findings in the original cohort.

**Table 2:** Incidences of cervical cancer by metformin exposure and hazard ratios comparing exposed to unexposed in the original cohort and the matched cohort, respectively

Metformin use	Case number	Incident cervical cancer	%	Person-years	Incidence rate (per 100,000 person-years)	Hazard ratio (95% confidence interval)	*P*
I. Original cohort							
Never users	6940	38	0.55	31307.79	121.38	1.000	
Ever users	132971	438	0.33	641413.41	68.29	0.558 (0.401-0.778)	0.0006
Tertiles of cumulative duration of metformin therapy (months)							
Never users	6940	38	0.55	31307.79	121.38	1.000	
<23.0	43778	254	0.58	161462.49	157.31	1.272 (0.904-1.790)	0.1679
23.0-47.9	44026	146	0.33	221949.93	65.78	0.523 (0.366-0.747)	0.0004
>47.9	45167	38	0.08	258001.00	14.73	0.109 (0.070-0.172)	<0.0001
II. Matched cohort							
Never users	6940	38	0.55	31303.68	121.39	1.000	
Ever users	6940	21	0.30	32891.65	63.85	0.522 (0.306-0.889)	0.0168
Tertiles of cumulative duration of metformin therapy (months)							
Never users	6940	38	0.55	31303.68	121.39	1.000	
<25.1	2287	12	0.52	8017.01	149.68	1.227 (0.639-2.355)	0.5383
25.1-50.4	2294	8	0.35	11443.50	69.91	0.562 (0.262-1.205)	0.1388
>50.4	2358	1	0.04	13431.15	7.45	0.061 (0.008-0.447)	0.0059

Cox regression models were created by incorporation with the inverse probability of treatment weighting using propensity score created from variables in Table 1 plus the entry date of the patients.

Tables 3 shows the overall hazard ratios in sensitivity analyses after excluding patients with various clinical conditions in the original cohort. Except for a non-significant *P* value in the model when users of sulfonylurea were excluded, all other models supported a significantly lower risk in ever users of metformin.

**Table 3:** Sensitivity analyses estimating hazard ratios for cervical cancer for ever vs. never users of metformin in the original cohort

Model	n/N in ever users	n/N in never users	HR (95% CI)	*P* value
I. Excluding patients who developed other cancers during follow-up	438 / 124945	38 / 6438	0.553 (0.397-0.771)	0.0005
II. Excluding patients who received cervical cancer screening	253 / 65095	26 / 3677	0.511 (0.341-0.765)	0.0011
III. Excluding users of sulfonylurea	149 / 48191	7 / 1888	0.753 (0.353-1.606)	0.4626
IV. Excluding users of insulin	427 / 130421	37 / 6459	0.535 (0.382-0.748)	0.0003
V. Excluding users of rosiglitazone	410 / 127111	37 / 6720	0.544 (0.388-0.761)	0.0004
VI. Excluding users of pioglitazone	420 / 130032	37 / 6769	0.557 (0.398-0.780)	0.0006

n: incident cases of cervical cancer, N: cases followed, HR: hazard ratio, CI: confidence interval

## DISCUSSION

This is the first study to suggest a significantly reduced risk of cervical cancer associated with metformin use. The reduced risk was not only observed in the overall analyses, but a dose-response pattern could also be seen in the tertile analyses (Table 2). The consistency in a well-matched cohort (Table 2) suggested that the conclusion was not affected by the imbalanced covariates in the original cohort (Table 1).

The mechanisms for a reduced risk of cervical cancer in metformin users remains to be explored. Chronic inflammation is a key component of cervical cancer progression [31]. Metformin reduces inflammation through improving metabolic disturbances or through inhibiting the proinflammatory cancer-promoting nuclear factor κB and STAT3 pathways [32]. Additionally, metformin inhibits the growth of cervical cancer cells through AMPK activation [33] or through an AMPK-independent pathway [26]. Metformin may exert an immune-mediated antitumor effect by increasing the number of CD8^+^ tumor-infiltrating lymphocytes [33]. It also impairs one-carbon metabolism and acts like an antifolate drug [34], and suppresses viral replication in hepatitis B [35] and C [36] infection (though whether similar effect can be observed in HPV infection is not known).

Competing risk of developing other cancers during follow-up did not affect the finding (Model I, Table 3). In addition, detection bias due to cervical cancer screening could not explain the lower risk in metformin users because a significantly higher proportion of them received such a screening in the original cohort (Table 1). If this could play a role, the overall hazard ratio suggesting a lower risk associated with metformin use in the original cohort (Table 2) would only be underestimated. It should also be pointed out that the finding after excluding patients who had received a screening program remained unaffected (Model II, Table 3).

The use of multiple antidiabetic drugs for glucose management may also affect the risk of cancer. For example, sulfonylurea, insulin, thiazolidinediones and incretin-based therapies have been implicated as potentially pro-tumorigenic [37–43]. Although most of them have been considered as potential confounders (Table 2) and have been evaluated by excluding users of them one at a time in modelling (Models III to VI, Table 3), it would be difficult to evaluate the interaction among these medications, especially when the frequent change of the drugs is taken into account. In the model after excluding users of sulfonylurea, the hazard ratio was not significant (Model III, Table 3). Therefore, the reduced risk in metformin users without excluding sulfonylurea (Table 2) could possibly be resulted from a residual confounding from sulfonylurea. However, the non-significant association after excluding sulfonylurea users (Model III, Table 3) could also be due to the lack of statistical power when a higher proportion of the patients had been excluded.

This study has several strengths related to the use of the nationwide databases of the NHI, which has been discussed previously [41, 44]. However, some limitations should be pointed out. First, HPV infection is an important risk factor [1], but we did not have such information. Second, obesity can be a risk factor of cancer [45] and body mass index is closely associated with cancer mortality [46]. However, we did not have anthropometric data for analyses. Third, we did not have biochemical data to evaluate their impact and there is a lack of information on the pathology, grading and staging of cervical cancer. Fourth, it is acknowledged that environmental factors and genetic disposition are all implicated in cancer development. Therefore, the interplay between family history, lifestyle, diet, and genetic parameters could not be evaluated. Finally, the observational nature is a major limitation. Because a comprehensive review and meta-analysis of randomized clinical trials did not support that metformin can reduce the risk of cancer [47], confirmation of the findings is certainly necessary.

In summary, this study is the first to show that metformin may significantly reduce the risk of cervical cancer, especially when it has been used for more than two years. However, future confirmation is mandatory.

## MATERIALS AND METHODS

The NHI reimbursement databases covering >99% of the Taiwan's residents have been described previously [41, 44]. They are handled by the National Health Research Institutes (NHRI) and can be used for academic researches if approved. The databases contain detailed records of every visit of each patient (including outpatient visits, emergency department visits and hospital admission) and include principal and secondary diagnostic codes, prescription orders, and claimed expenses.

Diabetes was coded 250.XX and cervical cancer 179-180, based on the *International Classification of Diseases, Ninth Revision, Clinical Modification* (ICD-9-CM).

Figure 1 shows the procedures in recruiting a cohort of female patients with newly diagnosed type 2 diabetes mellitus at an onset age of 25-74 years during the period from 1999 to 2005 (the original cohort). To assure that diabetes was first diagnosed after 1999, patients who had a diagnosis of diabetes mellitus during 1996-1998 were excluded. Patients should have been followed in the outpatient clinic with prescription of antidiabetic drugs for 2 or more times (*n*=423949). After a stepwise exclusion of ineligible patients, 139911 patients were recruited. Among them 132971 (95.04%) were ever treated with metformin and 6940 (4.96%) were never treated with metformin.

Cumulative duration (months) of metformin use was calculated and tertiles of cumulative duration were used for analyses. A number of comorbidities and covariates were included [48–50]: age, sex, hypertension (ICD-9-CM code: 401-405), chronic obstructive pulmonary disease (490-496), nephropathy (580-589), eye disease (250.5, 362.0, 369, 366.41 and 365.44), obesity (278), dyslipidemia (272.0-272.4), stroke (430-438), ischemic heart disease (410-414), and peripheral arterial disease (250.7, 785.4, 443.81 and 440-448). Other antidiabetic drugs included sulfonylurea, meglitinide, acarbose, insulin, pioglitazone and rosiglitazone. A history of receiving cervical cancer screening by Pap smear was also included as a potential confounder. Baseline characteristics were compared by Student's t test for age and by Chi-square test for the others.

The incidence density of cervical cancer was calculated for never users and ever users and for different subgroups of metformin exposure. Follow-up started on the first day of the use of antidiabetic drugs and ended on December 31, 2011, at the time of a new diagnosis of cervical cancer, or on the date of the last reimbursement record.

Logistic regression was used to create PS from all the baseline characteristics listed in Table 1 together with the entry date of each patient. The treatment effect was estimated by Cox regression incorporated with the IPTW using PS [29].

In consideration that the baseline characteristics were imbalanced between metformin ever and never users, additional analyses were conducted by using a 1:1 matched-pair sample (matched cohort) based on 8 digits of PS according to the methods described by Parsons [30]. Standardized differences were calculated using the methods described by Austin and Stuart [51]. A value of >10% might indicate meaningful imbalance with potential confounding [51].

In addition, the following models were created as sensitivity analyses in the original cohort by excluding: 1) patients who developed other cancers during follow-up; 2) patients who received cervical cancer screening; 3) users of sulfonylureas; 4) users of insulin; 5) users of rosiglitazone; and 6) users of pioglitazone.

Analyses were conducted using SAS statistical software, version 9.3 (SAS Institute, Cary, NC). *P*<0.05 was considered statistically significant.
